# Supplementary material for: Simultaneous invasion decouples zebra mussels and water clarity
Source: Commun Biol. 2022 Dec 22;5:1405. doi: 10.1038/s42003-022-04355-z (PMC9780222; doi:10.1038/s42003-022-04355-z)
Supplement: Supplementary file 2 — Supplementary Information [file 42003_2022_4355_MOESM2_ESM.pdf]

Supplementary Fig. 1. Partial dependence plots of individual covariates from the generalized additive model of water clarity in Fig. 3. The confidence band represents the 95% C. I. The invasion status categories for the AIS variable are Neither species present, ZM (*Dreissena* present), and ZM & SWF (*Dreissena* and *Bythotrephes* present).

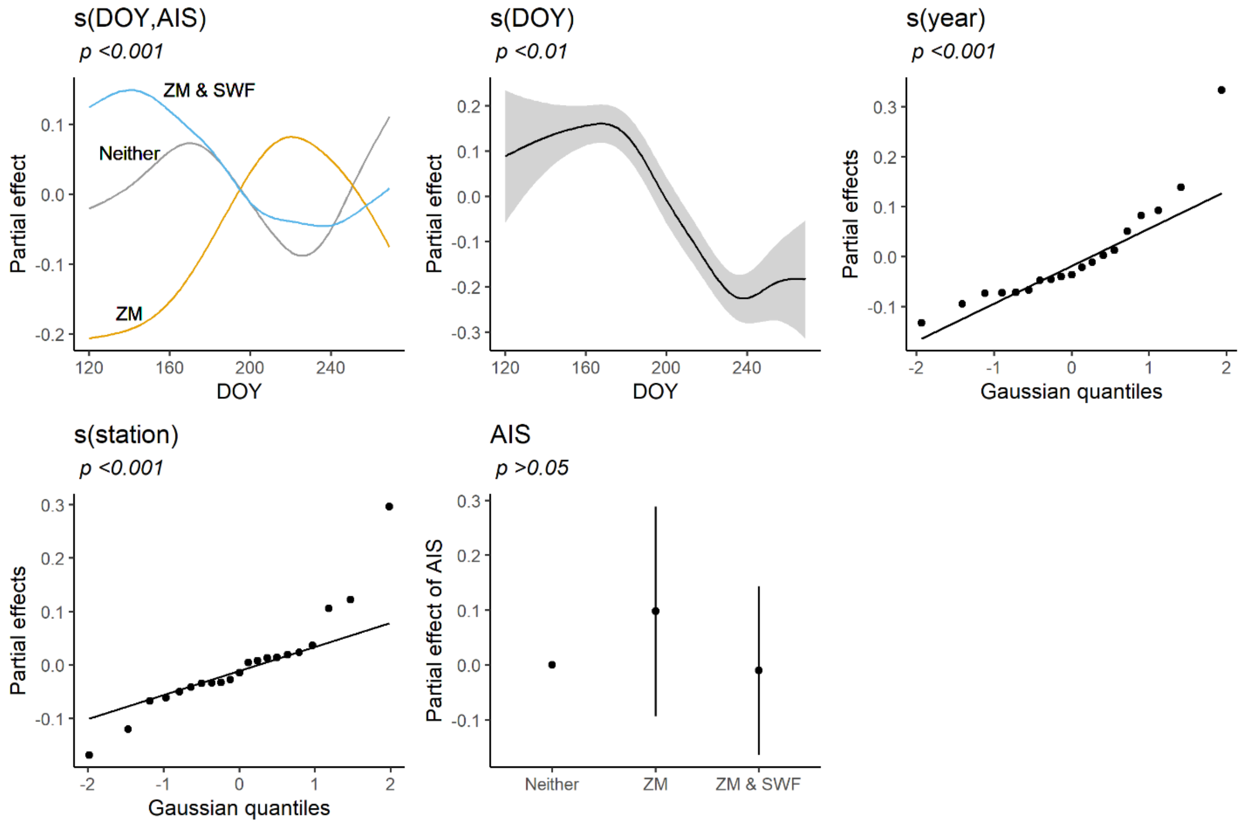

Supplementary Fig. 2. Plot of simulated water clarity (m) estimates at individual sampling locations versus different spatial metrics (n = 21 for each). Distance to ZM bed is the distance (m) to the nearest known *Dreissena* location based on diver surveys. Distance to shore (m) is the shortest straight-line distance between a sampling location and the shoreline.

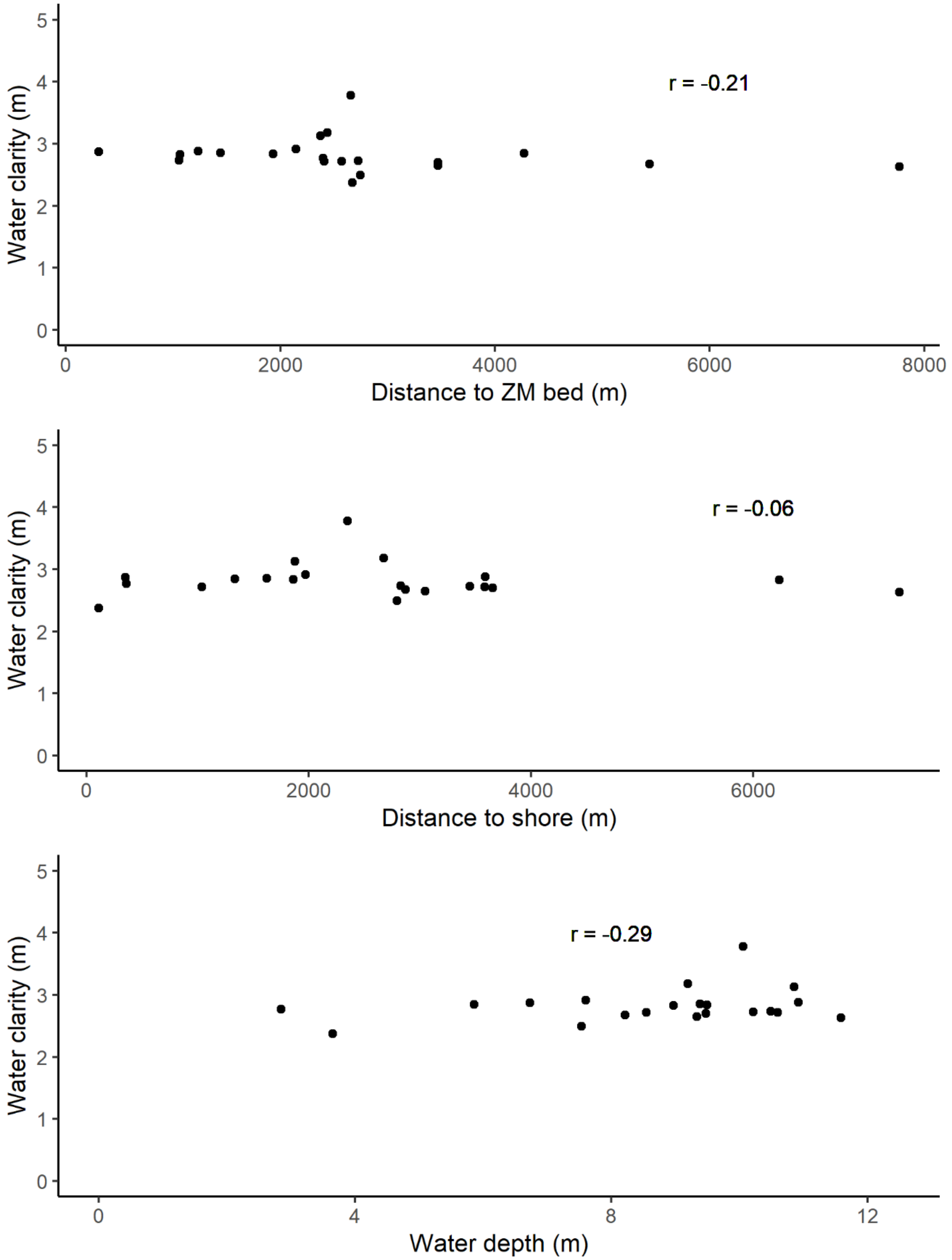

Supplementary Fig. 3. Land cover in the Lake Mille Lacs watershed, including the lake area, in 2001, 2006, 2011, and 2016, from the National Land Cover Database [75].

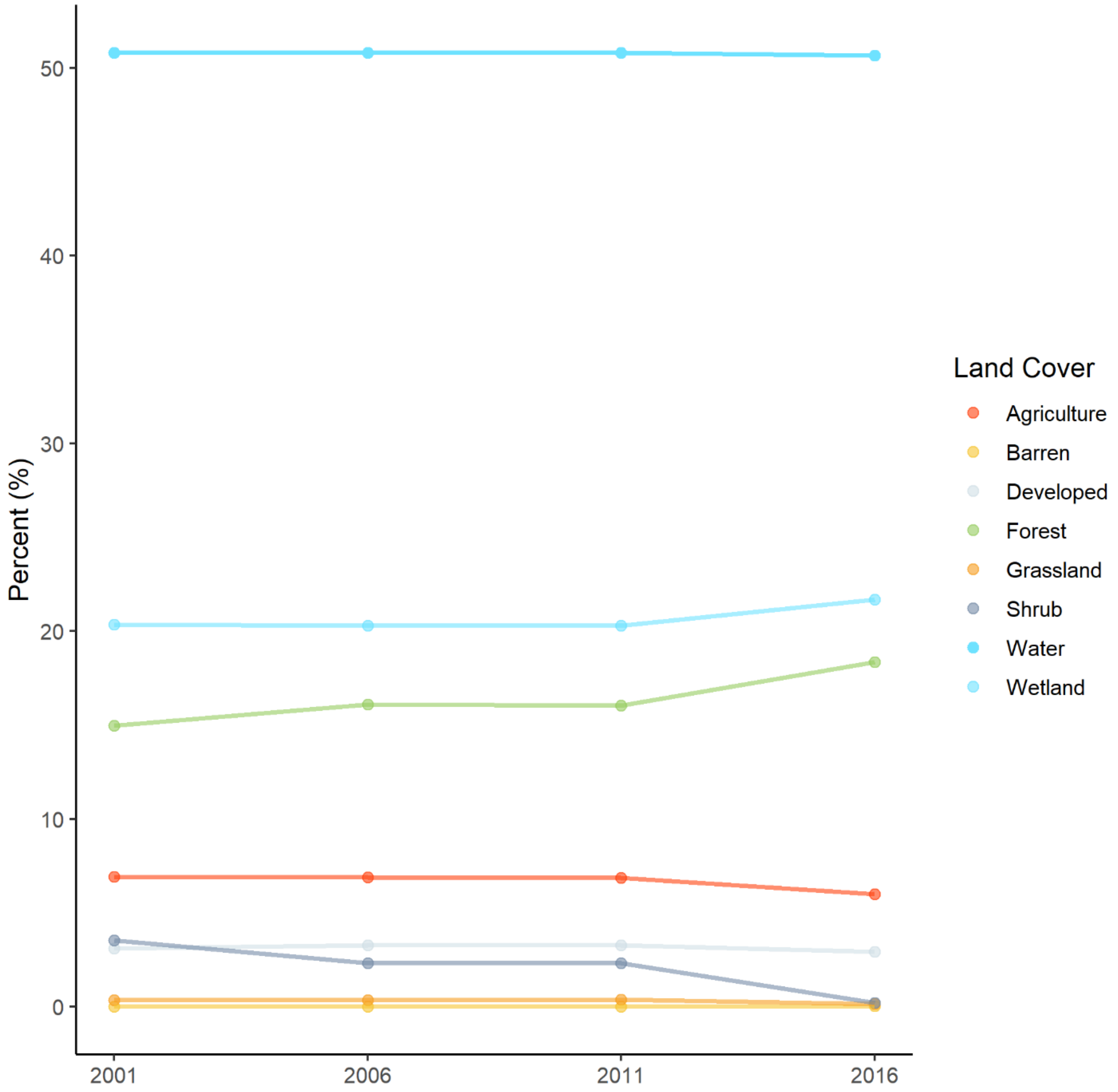

Supplementary Fig. 4. Water temperature characteristics. Upper, plot of cumulative degree days (base 0 °C) of water temperature at 2-m depth in Lake Mille Lacs from 2000-2018. Lower, box-plot of cumulative degree days (base 0 °C) by invasion status categories. The invasion status categories are Neither species present (n = 6), ZM (*Dreissena* present, n = 4), and ZM & SWF (*Dreissena* and *Bythotrephes* present, n = 9). The center line of the box-plots are the median, the 25$^{th}$-75$^{th}$ quartile are contained within the box, whiskers are 1.5x the interquartile range, and points are cumulative degree days for individual years.

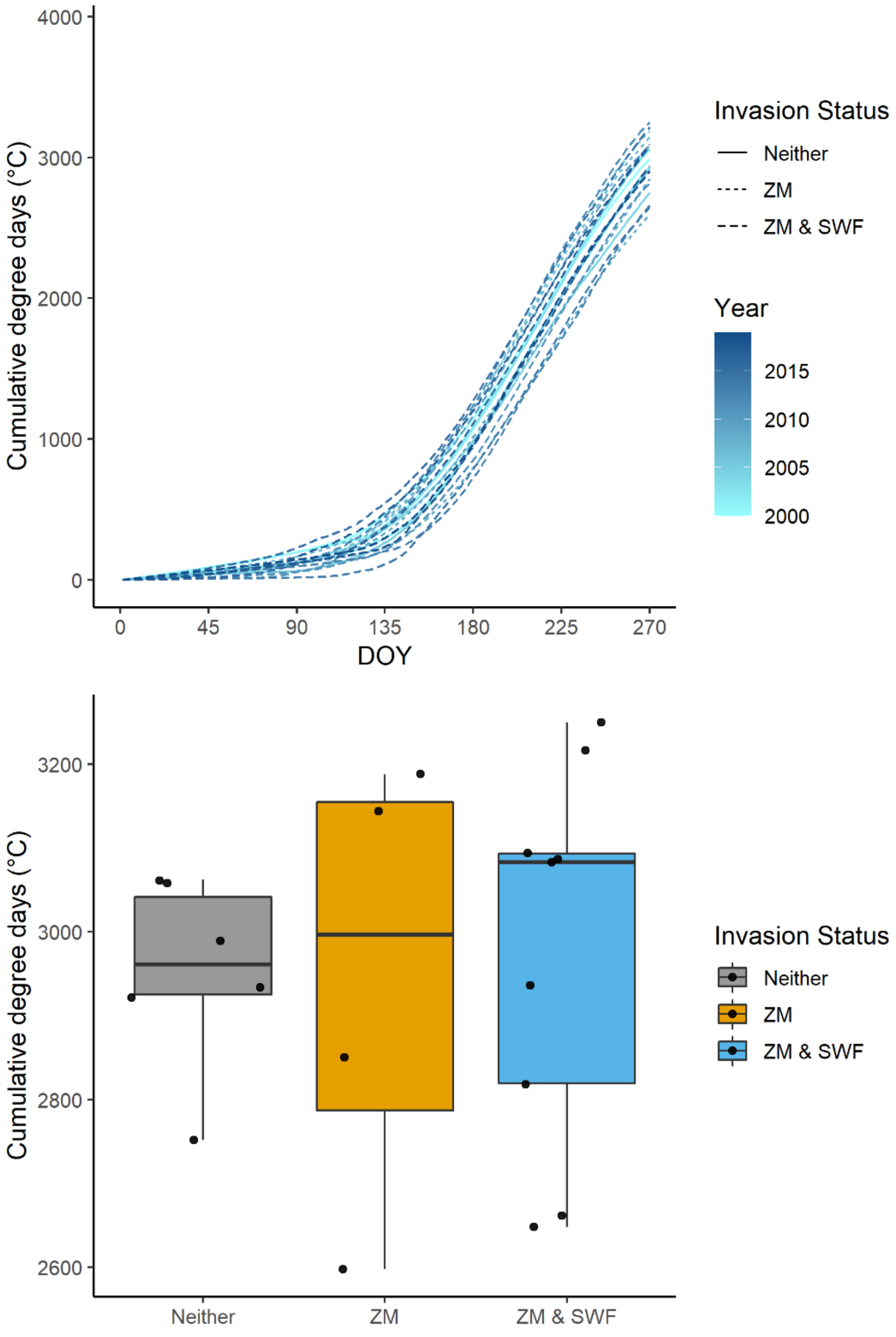

Supplementary Note 1

The following is code to reproduce the figures and supplementary figures in the manuscript using the program R.

```
library(dplyr)
library(ggplot2)
library(wesanderson)
library(gridExtra)
library(ggpubr)

#Figure 2, heatmap
pal<-wes_palette("Zissou1", 25, type="continuous")
p2013<-ggplot(data=filter(Tmap,yr==2013), aes(Date, Depth_m, z=temp)) +
  geom_tile(data=filter(Tmap,yr==2013), aes(fill = temp), show.legend = F, width=4)+
  stat_contour(binwidth=2)+
  scale_y_reverse(breaks=c(0,3,6,9))+
  scale_fill_gradientn(colours = (pal),limits=c(4,28))+
  scale_x_date(date_labels = "%b", date_breaks = "1 month")+
  labs(y="Depth (m)",x="")+
  ggtitle("2013")+
  theme_classic()
p2014<-ggplot(data=filter(Tmap,yr==2014), aes(Date, Depth_m, z=temp)) +
  geom_tile(data=filter(Tmap,yr==2014), aes(fill = temp), show.legend = F, width=4)+
  stat_contour(binwidth=2)+
  scale_y_reverse(breaks=c(0,3,6,9))+
  scale_fill_gradientn(colours = (pal),limits=c(4,28))+
  scale_x_date(date_labels = "%b", date_breaks = "1 month")+
  labs(y="",x="")+
  ggtitle("2014")+
  theme_classic()
p2015<-ggplot(data=filter(Tmap,yr==2015), aes(Date, Depth_m, z=temp)) +
  geom_tile(data=filter(Tmap,yr==2015), aes(fill = temp), show.legend = F, width=5)+
  stat_contour(binwidth=2)+
  scale_y_reverse(breaks=c(0,3,6,9))+
  scale_fill_gradientn(colours = (pal),limits=c(4,28))+
  scale_x_date(date_labels = "%b", date_breaks = "1 month")+
  labs(y="",x="")+
  ggtitle("2015")+
  theme_classic()+
  theme(axis.text.x=element_text(size = 9))
p2016<-ggplot(data=filter(Tmap,yr==2016), aes(Date, Depth_m, z=temp)) +
  geom_tile(data=filter(Tmap,yr==2016), aes(fill = temp), show.legend = F, width=4)+
  stat_contour(binwidth=2)+
```

```
  scale_y_reverse(breaks=c(0,3,6,9))+
  scale_fill_gradientn(colours = (pal),limits=c(4,28))+
  scale_x_date(date_labels = "%b", date_breaks = "1 month")+
  labs(y="Depth (m)",x="")+
  ggtitle("2016")+
  theme_classic()
p2017<-ggplot(data=filter(Tmap,yr==2017), aes(Date, Depth_m, z=temp)) +
  geom_tile(data=filter(Tmap,yr==2017), aes(fill = temp), show.legend = F, width=4)+
  stat_contour(binwidth=2)+
  scale_y_reverse(breaks=c(0,3,6,9))+
  scale_fill_gradientn(colours = (pal),limits=c(4,28))+
  scale_x_date(date_labels = "%b", date_breaks = "1 month")+
  labs(y="",x="")+
  ggtitle("2017")+
  theme_classic()
p2018<-ggplot(data=filter(Tmap,yr==2018), aes(Date, Depth_m, z=temp)) +
  geom_tile(data=filter(Tmap,yr==2018), aes(fill = temp), show.legend = F, width=4)+
  stat_contour(binwidth=2)+
  scale_y_reverse()+
  scale_fill_gradientn(colours = (pal),limits=c(4,28))+
  scale_x_date(date_labels = "%b", date_breaks = "1 month")+
  labs(y="",x="")+
  ggtitle("2018")+
  theme_classic()
p2019<-ggplot(data=filter(Tmap,yr==2019), aes(Date, Depth_m, z=temp)) +
  geom_tile(data=filter(Tmap,yr==2019), aes(fill = temp), show.legend = T, width=4)+
  stat_contour(binwidth=2)+
  scale_y_reverse()+
  scale_fill_gradientn(colours = (pal),limits=c(4,28), breaks=c(4,10,16,22,28))+
  scale_x_date(date_labels = "%b", date_breaks = "1 month")+
  labs(y="Depth (m)",x="", fill=expression("Temperature " ( degree~C)))+
  ggtitle("2019")+
  theme_classic()
legend1<-get_legend(p2019)
p2019<-ggplot(data=filter(Tmap,yr==2019), aes(Date, Depth_m, z=temp)) +
  geom_tile(data=filter(Tmap,yr==2019), aes(fill = temp), show.legend = F, width=4)+
  stat_contour(binwidth=2)+
  scale_y_reverse()+
  scale_fill_gradientn(colours = (pal),limits=c(4,28), breaks=c(4,10,16,22,28))+
  scale_x_date(date_labels = "%b", date_breaks = "1 month")+
  labs(y="Depth (m)",x="", fill=expression("Temperature " ( degree~C)))+
  ggtitle("2019")+
  theme_classic()
```

```
fig2<-grid.arrange(p2013, p2014,p2015, p2016, p2017,
                   p2018,p2019,legend1,nrow=3)

#Figure 3, Generalized Additive Model
fig3<-ggplot(nd1, aes(DOY, exp(mod_ig_fit)))+
  geom_line(aes(color=AIS, linetype=AIS),size=1,show.legend = FALSE)+
  geom_ribbon(aes(ymin=exp(mod_ig_fit-se.fit),
                  ymax=exp(mod_ig_fit+se.fit), fill=AIS), alpha=0.2, show.legend = F)+
  theme_classic(base_size = 12)+
  scale_color_manual(values=c("#999999","#E69F00","#56B4E9"))+
  scale_fill_manual(values=c("#999999","#E69F00","#56B4E9"))+
  labs(y="Clarity (m)", x="Day of Year")+
  scale_y_continuous(limits=c(0,6), breaks =seq(0,6,by=1))+
  scale_x_continuous(limits=c(120,270), breaks = seq(120,270,by=30))+
  geom_text(x=133,y=4.9, label="2010-2018", size=3.5)+
  geom_text(x=133,y=4.2, label="2000-2005", size=3.5)+
  geom_text(x=133,y=3.0, label="2006-2009", size=3.5)

#Figure 4, multiple time series plots
pd <- position_dodge(width = 0.2)
#ZM plot
p1<-ggplot()+
  geom_point(data=ZM2, aes(Year, Density), alpha=0.2, position = "jitter")+
  geom_point(data=ZM_boot2, aes(year, median.N),size=2)+
  theme_classic(base_size = 10)+
  labs(title="",x="",
       y =expression(atop(italic("D. polymorpha "),paste("(individuals " ~m^-2*")"))))+
  scale_x_continuous(breaks = c(2005, 2010, 2015, 2020), limits = c(2005, 2020),
                     labels=NULL)+
  scale_y_continuous(limits=c(0,25000))+
  geom_errorbar(data=ZM_boot2, aes(year, median.N,ymin=L.025, ymax=U.975), width=0.2)+
  geom_line(data=ZM_boot2, aes(year, median.N), show.legend = FALSE)+
  geom_text(data=ZM_boot2, aes(year, median.N),x=2019.5, y=15500, label="A", size=5)

#swf plot
p2<-ggplot(data=swf_boot)+
  geom_point(data=swf_boot,aes(year, median.N),
      color="black", size=2)+
  theme_classic(base_size = 10)+
  labs(title="",x="",
       y =expression(atop(italic("Bythotrephes "),paste("(individuals " ~m^-3*")"))))+
  scale_x_continuous(breaks = c(2005, 2010, 2015, 2020), limits = c(2005, 2020),
```

```
                    labels=NULL)+
  geom_errorbar(data=swf_boot,aes(year, median.N,ymin=L.025,
        ymax=U.975), color="black", width=0.2)+
  geom_line(data=swf_boot,aes(year, median.N),color="black")+
  geom_text(data=swf_boot,aes(year, median.N),x=2019.5, y=60, label="B", size=5)+
  geom_point(data=swf, aes(Year, swf_den), alpha=0.1, position = "jitter")

#zooplankton abundance plot
p3<-ggplot(data=zoop_boot)+
  geom_point(data=zoop_boot,aes(year, median.N,color=taxon,shape=taxon),
     size=2,position = pd,show.legend = FALSE)+
  theme_classic(base_size =10)+
  labs(title="",x="",
       y =expression(atop("Zooplankton", paste("(individuals "~L^-1*")"))))+
  scale_x_continuous(breaks = c(2005, 2010, 2015, 2020), limits = c(2005, 2020),
                    labels=NULL)+
  scale_y_continuous(breaks=c(0,25,50), limits=c(0,50))+
  geom_errorbar(data=zoop_boot,aes(year, median.N,color=taxon,
        ymin=L.025, ymax=U.975), width=0.2, position = pd,show.legend = FALSE)+
  geom_line(data=zoop_boot,aes(year, median.N,color=taxon,linetype=taxon),
           position = pd,show.legend = FALSE)+
  geom_text(data=zoop_boot,aes(year, median.N),x=2013.5, y=27, label="Copepods", size=3.5,
color="black")+
  geom_text(data=zoop_boot,aes(year, median.N),x=2008, y=7, label="Cladocerans", size=3.5,
color="black")+
  geom_text(data=zoop_boot,aes(year, median.N),x=2019.5, y=40, label="C", size=5,
color="black")+
  scale_shape_manual(values=c(16,16))+
  scale_color_manual(values=c("#993404","#fe9929"))+
  scale_linetype_manual(values=c("solid","solid"))+
  geom_point(data=zoop1, aes(year.x, cladoN), color="#993404", alpha=0.3,
            position="jitter")+
  geom_point(data=zoop1, aes(year.x, copeN), color="#fe9929", alpha=0.2,
          position="jitter")

#zoop production
p4<-ggplot(data=bythoP,aes(Year, X2oPdd))+
  geom_point(size=1.5, position = pd,show.legend = FALSE)+
  theme_classic(base_size = 10)+
  labs(title="",x="")+
  ylab(expression(atop("Bythotrephes P",paste("(\u03bcg "~m^-3*~dd^-1*")"))))+
  scale_x_continuous(breaks = c(2005, 2010, 2015, 2020), limits = c(2005, 2020),
                    labels=NULL)+
```

```
  scale_y_continuous(limits=c(0,55), breaks=c(0,10,20,30,40,50,60))+
  geom_errorbar(aes(ymin=L95CIdd, ymax=U95CIdd),
               position="dodge", width=0.1,show.legend = FALSE)+
  geom_line(show.legend = FALSE)+
  geom_text(x=2019.5, y=40, label="D", size=5, color="black")

p5<-ggplot(data=sum_zoopP,aes(year, Q50, color=taxon))+
  geom_point(aes(shape=taxon),size=1.5, position = pd,show.legend = FALSE)+
  theme_classic(base_size = 10)+
  labs(title="",x="")+
  ylab(expression(atop("Zooplankton P",paste("(\u03bcg "~L^-1*~dd^-1*")"))))+
  scale_x_continuous(breaks = c(2005, 2010, 2015, 2020), limits = c(2005, 2020),
                   labels=NULL)+
  scale_y_continuous(limits=c(0,5), breaks=c(0,1,2,3,4,5))+
  geom_errorbar(aes(ymin=Q2.5, ymax=Q97.5, color=taxon),
               position="dodge", width=0.25,show.legend = FALSE)+
  geom_line(aes(color=taxon, linetype=taxon),show.legend = FALSE)+
  scale_color_manual(values=c('#eb8055ff','#993404',"#f9b641ff"))+
  scale_shape_manual(values=c(16,16,16))+
  scale_linetype_manual(values=c("solid","solid","solid"))+
  geom_text(x=2009.6, y=3.75, label="Cyclopoids", size=3, color="black")+
  geom_text(x=2005.8, y=4, label="Clado-", size=3, color="black")+
  geom_text(x=2005.8, y=3, label="cerans", size=3, color="black")+
  geom_text(x=2012.6, y=3, label="Calanoids", size=3, color="black")+
  geom_text(x=2019.5, y=4, label="E", size=5, color="black")

#clarity and chlorophyll
p6<-ggplot(clarity)+
  geom_point(data=clarity, aes(year, median.N,color=parameter, shape=parameter),
            size=2, show.legend = FALSE, position=pd)+
  theme_classic(base_size = 10)+
  labs(title="",x="", y = (expression(atop("Chlorophyll-a",
   paste("(\u03bcg  "~L^-1*")")))))+
  scale_x_continuous(breaks = c(2005, 2010, 2015, 2020), limits = c(2005, 2020),
                   labels=NULL)+
  geom_errorbar(data=clarity,aes(year, median.N,ymin=(L.025),
                ymax=(U.975),color=parameter),
               width=0.2, show.legend = FALSE, position=pd)+
  geom_line(data=clarity,aes(year, median.N,color=parameter),
           show.legend = FALSE, position=pd)+
  scale_y_continuous(limits=c(0,25),sec.axis = sec_axis(~./4, name = "Clarity (m)"))+
  scale_color_manual(values = c("#a1dab4","#225ea8"))+
  scale_shape_manual(values=c(16,16,16))+
```

```
  geom_text(data=clarity,aes(year, median.N),x=2012, y=20, label="Clarity", size=3.5)+
  geom_text(data=clarity,aes(year, median.N),x=2015.5, y=5, label="Chl-a", size=3.5)+
  geom_text(data=clarity,aes(year, median.N),x=2019.5, y=15, label="F",
           size=5, color="black")+
  geom_point(data=secchi, aes(year, 4*d_secchi), color="#225ea8", alpha=0.1,
            position = "jitter")+
  geom_point(data=chloro2.1.1, aes(year, result), color="#a1dab4", alpha=0.1,
            position = "jitter")

#filter rates plot
p7<-ggplot(data=all.filter)+
  geom_point(aes(year, FzoopKm),color="#a8ddb5",size=2)+
  geom_line(aes(year, FzoopKm),color="#a8ddb5")+
  geom_point(aes(year, ZM_KM),color="#4eb3d3", size=2)+
  geom_line(aes(year, ZM_KM),color="#4eb3d3")+
  geom_point(aes(year, total_KM),color="#08589e", size=2)+
  geom_line(aes(year, total_KM), linetype="dashed",color="#08589e")+
  geom_errorbar(aes(year,
FzoopKm,ymin=(FzoopKM_L95),ymax=(FzoopKM_U95)),color="#a8ddb5",
               width=0.2, show.legend = FALSE, position=pd)+
  geom_errorbar(aes(year, ZM_KM,ymin=(L95CI.x),ymax=(U95CI.x)),color="#4eb3d3",
               width=0.2, show.legend = FALSE, position=pd)+
  geom_errorbar(aes(year, total_KM,ymin=(total_L95),ymax=(total_U95)),color="#08589e",
               width=0.2, show.legend = FALSE, position=pd)+
  theme_classic(base_size = 10)+
  labs(title="",x="", y = "Filtering Rate ("~km^3*~d^-1*")")+
  scale_x_continuous(breaks = c(2005, 2010, 2015, 2020), limits = c(2005, 2020),labels =
NULL)+
  geom_text(x=2007, y=0.5, label="Zebra Mussels", size=3.5)+
  geom_text(x=2015.5, y=0.5, label="Zooplankton", size=3.5)+
  geom_text(x=2015, y=2, label="Total", size=3.5)+
  geom_text(x=2019.5, y=2, label="G", size=5, color="black")

#TP plot
p8<-ggplot(data=TP2,aes(year, TP_yr))+
  geom_point(data=TP2,aes(year, TP_yr),size=1.5, position = pd)+
  theme_classic(base_size = 10)+
  labs(title="",x="")+
  ylab(expression(atop("Total P (mg "~L^-1*")")))+
  scale_x_continuous(breaks = c(2005,2010, 2015,2020),
                     limits = c(2005, 2020), labels = NULL)+
  scale_y_continuous(limits=c(0,0.08), breaks=c(0,.02,.04,.06,.08))+
  geom_errorbar(data=TP2,aes(year, TP_yr,ymin=TP_yr-TP_se, ymax=TP_yr+TP_se),
```

```
              position="dodge", width=0.1,show.legend = FALSE)+
  geom_line(data=TP2,aes(year, TP_yr),show.legend = FALSE)+
  geom_text(data=TP2,aes(year, TP_yr),x=2019.5, y=0.06, label="H",
    size=5, color="black")+
  geom_point(data=TP1, aes(year, Result), alpha=0.1,position = "jitter")


#fish
p9<-ggplot()+
  geom_point(data=filter(fish1, species=="YEP"), aes(year, CPU,shape=location),
             alpha=0.2,color="#edae49",show.legend = FALSE,position = "jitter")+
  geom_point(data=filter(fish2, species=="YEP"),aes(year, cpue,
            shape=location),size=2,
             color="#edae49",show.legend = FALSE)+
  geom_line(data=filter(fish2, species=="YEP"),aes(year, cpue,
            linetype=location),color="#edae49",
            show.legend = FALSE)+
  theme_classic(base_size = 10)+
  labs(title="",x="", y = "Yellow Perch (CPU)")+
  scale_x_continuous(breaks = c(2005, 2010, 2015, 2020),
                     limits = c(2005, 2020),labels=NULL)+
  scale_y_continuous(limits=c(0, 100))+
  geom_errorbar(data=filter(fish2, species=="YEP"),aes(year, cpue,
              ymin=(cpue-se),ymax=(cpue+se)),color="#edae49",
              width=0.2, show.legend = FALSE, position=pd)+
  geom_text(data=filter(fish2, species=="YEP"),aes(year, cpue),x=2019.5, y=80,
    label="I", size=5, color="black")+
  scale_linetype_manual(values=c("solid","dashed"))

p10<-ggplot()+
  geom_point(data=filter(fish1, species=="TLC"), aes(year, CPU,shape=location),
             alpha=0.2,color="#00798c",show.legend = FALSE,position = "jitter")+
  geom_point(data=filter(fish2, species=="TLC"),aes(year, cpue,shape=location),
             size=2,color="#00798c",show.legend = FALSE)+
  geom_line(data=filter(fish2, species=="TLC"),aes(year, cpue, linetype=location),
            show.legend = FALSE, color="#00798c")+
  theme_classic(base_size = 10)+
  labs(title="",x="", y = "Cisco (CPU)")+
  scale_x_continuous(breaks = c(2005, 2010, 2015, 2020),
                     limits = c(2005, 2020))+
  scale_y_continuous(limits=c(0, 125))+
  geom_errorbar(data=filter(fish2, species=="TLC"),
              aes(year, cpue, ymin=(cpue-se),ymax=(cpue+se)),
```

```
                width=0.2, show.legend = FALSE, position=pd,color="#00798c")+
  geom_text(data=filter(fish2, species=="TLC"),aes(year, cpue),
            x=2019.5, y=100, label="J", size=5, color="black")+
  scale_linetype_manual(values=c("solid","dashed"))

#zoop size
p11<-ggplot()+
  geom_point(data=size_yr2, aes(year, length_microns),
             alpha=0.1,shape=1,color="goldenrod", position="jitter")+
  geom_point(data=size_yr,aes(x=year, y=year_L), color="goldenrod4")+
  geom_line(data=size_yr,aes(x=year, y=year_L), color="goldenrod4")+
  geom_errorbar(data=size_yr,aes(x=year, y=year_L,ymin=year_L-year_se,
                              ymax=year_L+year_se),
                width=0.2, position = pd,show.legend = FALSE, color="goldenrod4")+
  theme_classic(base_size=10)+
  labs(title="",x="",
       y =expression(atop("Zooplankton Length", paste("(\u03bcm)"))))+
  scale_x_continuous(breaks = c(2005, 2010, 2015, 2020), limits = c(2005, 2020))+
  geom_text(data=size_yr, aes(x=year, y=year_L),x=2019.5, y=3000, label="K",
            size=5, color="black")

#combine all plots
fig4 <-ggarrange(p1, p2,p3, p4, p6, p7,
                 p8, p9, p10, p11,
                 ncol=2,nrow=6,align = "v")

#Figure 5, Predator-prey plots
p12<-ggplot(zoop_d,aes(swf_d,clado_d))+
  geom_point(color='#993404')+
  theme_classic()+
  labs(x=expression(~italic(Bythotrephes)~" (individuals " ~m^-3*")"),
       y="Cladocerans (individuals " ~L^-1*")")
p13<-ggplot(zoop_d,aes(swf_d,cala_d))+
  geom_point(color="#eb8055ff")+
  theme_classic()+
  labs(x=expression(~italic(Bythotrephes)~" (individuals " ~m^-3*")"),
       y="Calanoids (individuals " ~L^-1*")")
p14<-ggplot(zoop_d,aes(swf_d,cyclo_d))+
  geom_point(color="#f9b641ff")+
  theme_classic()+
  labs(x=expression(~italic(Bythotrephes)~" (individuals " ~m^-3*")"),
       y="Cyclopoids (individuals " ~L^-1*")")
p15<-ggplot(zoop_fish)+
```

```
  geom_point(aes(YEP_nearshore,clado_y),color='#993404',shape=16)+
  geom_point(aes(YEP_offshore,clado_y),color='#993404',shape=1)+
  theme_classic()+
  labs(x="Yellow Perch (CPU)",
       y="Cladocerans (individuals " ~L^-1*")")
p16<-ggplot(zoop_fish)+
  geom_point(aes(YEP_nearshore,cala_y),color="#eb8055ff",shape=16)+
  geom_point(aes(YEP_offshore,cala_y),color="#eb8055ff",shape=1)+
  theme_classic()+
  labs(x="Yellow Perch (CPU)",
       y="Calanoids (individuals " ~L^-1*")")
p17<-ggplot(zoop_fish)+
  geom_point(aes(YEP_nearshore,cyclo_y),color='#f9b641ff',shape=16)+
  geom_point(aes(YEP_offshore,cyclo_y),color='#f9b641ff',shape=1)+
  theme_classic()+
  labs(x="Yellow Perch (CPU)",
       y="Cyclopoids (individuals " ~L^-1*")")
p18<-ggplot(zoop_fish)+
  geom_point(aes(TLC_nearshore,clado_y),color='#993404',shape=16)+
  geom_point(aes(TLC_offshore,clado_y),color='#993404',shape=1)+
  theme_classic()+
  labs(x="Cisco (CPU)",
       y="Cladocerans (individuals " ~L^-1*")")
p19<-ggplot(zoop_fish)+
  geom_point(aes(TLC_nearshore,cala_y),color="#eb8055ff",shape=16)+
  geom_point(aes(TLC_offshore,cala_y),color="#eb8055ff",shape=1)+
  theme_classic()+
  labs(x="Cisco (CPU)",
       y="Calanoids (individuals " ~L^-1*")")
p20<-ggplot(zoop_fish)+
  geom_point(aes(TLC_nearshore,cyclo_y),color='#f9b641ff',shape=16)+
  geom_point(aes(TLC_offshore,cyclo_y),color='#f9b641ff',shape=1)+
  theme_classic()+
  labs(x="Cisco (CPU)",
       y="Cyclopoids (individuals " ~L^-1*")")

fig5<-ggarrange(p12, p13,p14,p15,p16,p17,p18,p19,p20,
                ncol=3,nrow=3,align = "v")

#Figure 6, pre- and post-invasion characteristics
fig6<-ggplot(change.df2,aes(taxon,median.N, fill=group1))+
  geom_bar(stat="identity",color="black",position=position_dodge())+
  geom_errorbar(aes(ymin=(L.025),ymax=(U.975)),
```

```
                width=0.2, show.legend = FALSE, position=position_dodge(0.9))+
  theme_classic(base_size = 15)+
  labs(y="", x="", fill="")+
  scale_x_discrete(labels=c("Clarity"="Clarity (m)",
                            "Chlorophyll-a"="Chlorophyll-a (\u03BCg" ~L^-1*")",
                            "Total Filtering"=expression("Total Filtering ("*km^3*~d^-1*")"),
                            "Copepod"="Copepods (individuals" ~L^-1*")",
                            "Cladoceran"="Cladocerans (individuals" ~L^-1*")",
                            "Bythotrephes"=expression(paste(italic(Bythotrephes)," (individuals" ~m^-
3*")")),
                            "Dreissenax1000"=expression(paste(italic(D.~polymorpha),"
1000x(individuals" ~m^-2*")"))))+
  coord_flip()+
  scale_fill_manual(values = c("#FFD662FF","#00539CFF"), labels=c("2011-2018","2006-
2010"))+
  guides(fill=guide_legend(reverse=TRUE))+
  theme(legend.position = c(.8,.2),legend.text=element_text(size=15),
        legend.key.height =unit(1,'cm'),legend.key.width= unit(1,'cm'))+
  annotate(geom="text", x=6, y=20, label="*", size=10)+
  annotate(geom="text", x=7, y=20, label="*", size=10)+
  annotate(geom="text", x=4.9, y=30, label="*", size=10)+
  annotate(geom="text", x=3.9, y=40, label="*", size=10)

#Supplementary Figure 1, GAM individual smooths
p21<-ggplot(doy_ais, aes(DOY,est))+
  geom_line(aes(color=as.factor(AIS)),show.legend = FALSE)+
  theme_classic()+
  theme(legend.position = "none")+
  scale_color_manual(values=c("#999999","#E69F00","#56B4E9"))+
  geom_text(x=180,y=0.15, label="ZM & SWF", size=3.5, color="black")+
  geom_text(x=140,y=0.06, label="Neither", size=3.5, color="black")+
  geom_text(x=140,y=-0.17, label="ZM", size=3.5, color="black")+
  labs(title="s(DOY,AIS)",subtitle = ~italic("p <0.001"),
       x="DOY", y="Partial effect")
p22<-ggplot(doy, aes(DOY,est))+
  geom_ribbon(aes(ymin=est-se,ymax=est+se), fill="lightgray")+
  geom_line(color="black")+
  theme_classic()+
  labs(title="s(DOY)",subtitle = ~italic("p <0.01"),
       x="DOY", y="Partial effect")
p23<-ggplot(fyear, aes(sample=est))+
         stat_qq()+
         stat_qq_line()+
```

```
  theme_classic()+
  labs(title="s(year)",subtitle = ~italic("p <0.001"),
       x="Gaussian quantiles", y="Partial effects")
p24<-ggplot(station, aes(sample=est))+
  stat_qq()+
  stat_qq_line()+
  theme_classic()+
  labs(title="s(station)",subtitle = ~italic("p <0.001"),
       x="Gaussian quantiles", y="Partial effects")
p25<-ggplot(ais, aes(as.factor(value),partial))+
  geom_point()+
  geom_errorbar(aes(ymin=partial-(2*se),ymax=partial+(2*se)),width=0)+
  theme_classic()+
  scale_x_discrete(labels=c("Neither","ZM", "ZM & SWF"))+
  labs(x="",title="AIS",subtitle = ~italic("p >0.05"), y="Partial effect of AIS")

figSI1<-ggarrange(p21,p22,p23,p24,p25,ncol=3,nrow=2)

#Supplementary Figure 2, Spatial patterns in water clarity
p26<-ggplot(nd2, aes(dist_ZM,fit))+
  geom_point()+
  theme_classic()+
  scale_y_continuous(limits=c(0,5))+
  labs(x="Distance to ZM bed (m)",y="Water clarity (m)")+
  geom_text(x=6000,y=4, label="r = -0.21", size=3.5, color="black")
p27<-ggplot(nd2, aes(dist_shore,fit))+
  geom_point()+
  theme_classic()+
  scale_y_continuous(limits=c(0,5))+
  labs(x="Distance to shore (m)",y="Water clarity (m)")+
  geom_text(x=6000,y=4, label="r = -0.06", size=3.5, color="black")
p28<-ggplot(nd2, aes(-depth,fit))+
  geom_point()+
  theme_classic()+
  scale_y_continuous(limits=c(0,5),)+
  scale_x_continuous(limits=c(0,12.5))+
  labs(x="Water depth (m)",y="Water clarity (m)")+
  geom_text(x=8,y=4, label="r = -0.29", size=3.5, color="black")

figSI2<-ggarrange(p26, p27,p28,
                  ncol=1,nrow=3,align = "v")

#Supplementary Figure 3, Land cover trends
```

```
figSI3<-ggplot(land, aes(year,per))+
  geom_point(aes(color=landuse), size=2)+
  geom_line(aes(color=landuse),show.legend = F,size=1)+
  theme_classic(base_size = 12)+
  scale_x_continuous(limits=c(2001,2016), breaks=c(2001,2006,2011,2016))+
  labs(x="", y="Percent (%)", color="Land Cover")+
  scale_color_manual(values=c("#FF410D99", "#F7C53099",
  "#D0DFE699","#95CC5E99","#F79D1E99","#748AA699","#6EE2FFFF","#6EE2FF99"))

# Supplementary Figure 4, Water temperature characteristics
dd1<-ggplot(temp, aes(DOY, dd.cum, group=Year))+
  geom_line(aes(color = Year, linetype=ais))+
  theme_classic(base_size = 12)+
  scale_x_continuous(limits=c(0,270), breaks=c(0,45,90,135,180,225,270))+
  labs(y="Cumulative degree days (\u00b0C)")+
  scale_color_gradient(low = 'darkslategray1', high = 'dodgerblue4')+
  labs(linetype="Invasion Status")

#look at cumulative dd on day 270
temp2<-temp%>%filter(DOY==270&Year<2019)
dd2<-ggplot(temp2, aes(ais, dd.cum, fill=ais))+
  geom_boxplot()+
  geom_jitter(color="black", size=1.5, alpha=0.9)+
  theme_classic(base_size = 12)+
  scale_fill_manual(values=c("#999999", "#E69F00", "#56B4E9"))+
  labs(fill="Invasion Status", y="Cumulative degree days (\u00b0C)",x="")

FigSI4<-ggarrange(dd1,dd2,nrow = 2,ncol = 1)
```
