# Supplementary material for: Simultaneous invasion decouples zebra mussels and water clarity
Source: Commun Biol. 2022 Dec 22;5:1405. doi: 10.1038/s42003-022-04355-z (PMC9780222; doi:10.1038/s42003-022-04355-z)
Supplement: Supplementary file 3 — Description of Additional Supplementary Files [file 42003_2022_4355_MOESM3_ESM.pdf]

Description of Additional Supplementary Files

File name: Supplementary Data
Description: Text files used to create figures (33 files total).
