# Supplementary material for: Simultaneous invasion decouples zebra mussels and water clarity
Source: Commun Biol. 2022 Dec 22;5:1405. doi: 10.1038/s42003-022-04355-z (PMC9780222; doi:10.1038/s42003-022-04355-z)
Supplement: Supplementary file 5 — Reporting Summary [file 42003_2022_4355_MOESM5_ESM.pdf]

nature portfolio

Corresponding author(s): Heidi M. Rantala

Last updated by author(s): 10/28/2022

Reporting Summary

Nature Portfolio wishes to improve the reproducibility of the work that we publish. This form provides structure for consistency and transparency in reporting. For further information on Nature Portfolio policies, see our Editorial Policies and the Editorial Policy Checklist.

Statistics

For all statistical analyses, confirm that the following items are present in the figure legend, table legend, main text, or Methods section.

n/a	Confirmed	
☐	☒	The exact sample size (*n*) for each experimental group/condition, given as a discrete number and unit of measurement
☐	☒	A statement on whether measurements were taken from distinct samples or whether the same sample was measured repeatedly
☐	☒	The statistical test(s) used AND whether they are one- or two-sided<br>*Only common tests should be described solely by name; describe more complex techniques in the Methods section.*
☐	☒	A description of all covariates tested
☐	☒	A description of any assumptions or corrections, such as tests of normality and adjustment for multiple comparisons
☐	☒	A full description of the statistical parameters including central tendency (e.g. means) or other basic estimates (e.g. regression coefficient) AND variation (e.g. standard deviation) or associated estimates of uncertainty (e.g. confidence intervals)
☐	☒	For null hypothesis testing, the test statistic (e.g. *F*, *t*, *r*) with confidence intervals, effect sizes, degrees of freedom and *P* value noted<br>*Give P values as exact values whenever suitable.*
☒	☐	For Bayesian analysis, information on the choice of priors and Markov chain Monte Carlo settings
☐	☒	For hierarchical and complex designs, identification of the appropriate level for tests and full reporting of outcomes
☒	☐	Estimates of effect sizes (e.g. Cohen's *d*, Pearson's *r*), indicating how they were calculated

Our web collection on statistics for biologists contains articles on many of the points above.

Software and code

Policy information about availability of computer code

Data collection	Neither software nor code was used to collect data for this study.
Data analysis	Data summaries and filtering rate calculations were conducted in R using custom code, with the exception of the secondary production estimates, which were calculated using code modified from Cross et al. (2010). Code for Figs. 2-5 and Supplementary Figs. 1-4 are included as Supplementary Text. Other computer codes used for this work are available from the corresponding author upon request.

For manuscripts utilizing custom algorithms or software that are central to the research but not yet described in published literature, software must be made available to editors and reviewers. We strongly encourage code deposition in a community repository (e.g. GitHub). See the Nature Portfolio guidelines for submitting code & software for further information.

Data

Policy information about availability of data

All manuscripts must include a data availability statement. This statement should provide the following information, where applicable:

- Accession codes, unique identifiers, or web links for publicly available datasets
- A description of any restrictions on data availability
- For clinical datasets or third party data, please ensure that the statement adheres to our policy

Fish, lake water chemistry, zebra mussel, zooplankton, and water temperature data are available from the corresponding author on reasonable request. Water clarity data are available in the Minnesota Pollution Control Agency (MPCA) Surface Water Data repository with the unique waterbody identifier

"48-0002-00" [https://webapp.pca.state.mn.us/surface-water/search?dataType=All%20Stations&wid=48-0002-00]. Landuse data are available from the National Land Cover Database (www.usgs.gov). Other spatial data are available from the Minnesota Geospatial Commons (www.gisdata.mn.gov). Modelled water temperature data, which we used to impute missing values from water temperature data collected by sensors, are available from the United States Geological Survey ScienceBase Catalog (www.sciencebase.gov). Figs. 2, 4, 5, 6, and Supplementary Figs. 2-4 use data from the sources listed above. Data for Figs. 2 - 6 and Supplementary Figs. 1- 4 are available in the Supplementary Data.

Human research participants

Policy information about studies involving human research participants and Sex and Gender in Research.

Reporting on sex and gender	*Use the terms sex (biological attribute) and gender (shaped by social and cultural circumstances) carefully in order to avoid confusing both terms. Indicate if findings apply to only one sex or gender; describe whether sex and gender were considered in study design whether sex and/or gender was determined based on self-reporting or assigned and methods used. Provide in the source data disaggregated sex and gender data where this information has been collected, and consent has been obtained for sharing of individual-level data; provide overall numbers in this Reporting Summary. Please state if this information has not been collected. Report sex- and gender-based analyses where performed, justify reasons for lack of sex- and gender-based analysis.*
Population characteristics	*Describe the covariate-relevant population characteristics of the human research participants (e.g. age, genotypic information, past and current diagnosis and treatment categories). If you filled out the behavioural & social sciences study design questions and have nothing to add here, write "See above."*
Recruitment	*Describe how participants were recruited. Outline any potential self-selection bias or other biases that may be present and how these are likely to impact results.*
Ethics oversight	*Identify the organization(s) that approved the study protocol.*

Note that full information on the approval of the study protocol must also be provided in the manuscript.

Field-specific reporting

Please select the one below that is the best fit for your research. If you are not sure, read the appropriate sections before making your selection.

☐ Life sciences ☐ Behavioural & social sciences ☒ Ecological, evolutionary & environmental sciences

For a reference copy of the document with all sections, see nature.com/documents/nr-reporting-summary-flat.pdf

Ecological, evolutionary & environmental sciences study design

All studies must disclose on these points even when the disclosure is negative.

Study description	This study utilized an existing long-term dataset collected by state agency biologists to explore hypotheses related to the simultaneous invasion of two invertebrate species in a north temperature freshwater lake. We present a 13-year dataset representing multiple trophic levels and their trends through time, including observations and modeled values.
Research sample	Water clarity samples were included as the lake characteristic of interest because it is a often used metric of lake health and is known to change due to zebra mussels. Zebra mussels were sampled to monitor their population size, as they represent an invasive species known to have large effects on freshwater ecosystems, especially to water clarity. The zooplankton community was sampled as an indicator of food web change that responds relatively quickly to perturbations and may have top-down effects on water clarity and compete with zebra mussels for food resources. Water chemistry data from Lake Mille Lacs were sampled to test the potential for bottom-up effects to buffer the effects of zebra mussels. Planktivorous fish were collected as part of a long-term monitoring effort used to inform fisheries management and utilized here to test if their populations could have top-down effects on water clarity. Land cover data were included to demonstrate that significant land use changes occurred in the watershed that would drive bottom-up effects on water clarity by nutrient addition.
Sampling strategy	A sample size calculation was not used in this work. Here, we presented long term data from a monitoring program that were collected for agency use and included data from years when all trophic levels were sampled (2006-2018).
Data collection	No new data were collected for this work. Agency biologists collected and recorded field and laboratory data using data applications and programs. Non-scientific community monitoring data were entered to the MPCA through a web portal. Chemistry data were collected by the Minnesota Department of Agriculture and Minnesota Department of Health laboratories, which are certified entities.
Timing and spatial scale	Lake water chemistry and zooplankton sampling occurred monthly from May to September in 2006-2018 as part of a routine monitoring program. Fish and zebra mussel sampling occurred annual from 2006-2018. Land cover data for the watershed were collected in 2001, 2006, 2011, and 2016 by USGS. Water clarity data were collected by multiple state and tribal agencies, as well as through a community science program at irregular intervals from 2000-2018.
Data exclusions	No data were excluded from these analyses.

Reproducibility	Modeled values and data summaries are reproducible using the dataset and code. The reproducibility of the dataset through the collection of a parallel observational study is not logistically feasible, given the coincidental simultaneous population growth of two invasive species during an existing monitoring program.
Randomization	Randomization of groups is not applicable to this work, as the study was not replicated or experimental.
Blinding	Blinding was not possible this study, as we analysed previously collected data from a long-term monitoring program that was collected for other purposes. We did not perform analyses that would be suitable for blinding.

Did the study involve field work? ☒ Yes ☐ No

Field work, collection and transport

Field conditions	Fieldwork was conducted from May-September when sampling conditions were safe. Unsafe conditions in this system arise during lightning storms and moderate to high winds. Air temperatures at the study location varied from 10-25 C during May-September from 2006-2018.
Location	The work was conducted at Lake Mille Lacs in Minnesota, USA, latitude 46.26, longitude -93.58 , elevation ~ 380 m. Zooplankton tows were taken from the entire water column. Lake water chemistry samples were collected from the top 2 m of the lake surface. Gill nets were set with the lead line on the lake bottom, once a year, at 20 nearshore and 32 offshore locations. Water clarity data were collected at 21 locations around the lake.
Access & import/export	Permits were not required for this work, as it was conducted by state agencies for management purposes and did not include threatened or endangered species or movement across state or international borders.
Disturbance	This study did not cause disturbance.

Reporting for specific materials, systems and methods

We require information from authors about some types of materials, experimental systems and methods used in many studies. Here, indicate whether each material, system or method listed is relevant to your study. If you are not sure if a list item applies to your research, read the appropriate section before selecting a response.

Materials & experimental systems

n/a	Involved in the study	
☒	☐	Antibodies
☒	☐	Eukaryotic cell lines
☒	☐	Palaeontology and archaeology
☐	☒	Animals and other organisms
☒	☐	Clinical data
☒	☐	Dual use research of concern

Methods

n/a	Involved in the study	
☒	☐	ChIP-seq
☒	☐	Flow cytometry
☒	☐	MRI-based neuroimaging

Animals and other research organisms

Policy information about studies involving animals; ARRIVE guidelines recommended for reporting animal research, and Sex and Gender in Research

Laboratory animals	This study did not involve laboratory animals.
Wild animals	Fish were collected using gill nets. As nets were set for ~ 20 hours, most fish died in the process and were processed for collection of demographic information (length, mass, aging structure). Live fish are returned to the lake if possible, after length and mass was collected for that individual. If a live fish was not returned to the lake, it was dispatched via pithing or cervical displacement. Zooplankton were dispatched immediately by placing them in 80% ethanol.
Reporting on sex	Sex of organisms was not considered in the study design.
Field-collected samples	This study did not involve laboratory work with live field collected animals.
Ethics oversight	No ethical approval was needed for this work, as data were collected by natural resource biologists for management purposes. Biologists, however, follow the guidelines outlined in "Guidelines for the Use of Fishes in Research" which was co-published by the American Fisheries Society, the American Institute of Fishery Research Biologists, and the American Society of Ichthyologists and Herpetologists.

Note that full information on the approval of the study protocol must also be provided in the manuscript.
